# Abdominal Aortic Aneurysms in Two Prosected Anatomical Donors: Correlations With Aortoiliac Occlusive Disease and Pelvic Congestion Syndrome

**DOI:** 10.7759/cureus.77489

**Published:** 2025-01-15

**Authors:** Nicole L Geske, Elena O Watson, Apoorva Dayananda, William McMillan

**Affiliations:** 1 Radiology, Division of Human Anatomy, Michigan State University College of Osteopathic Medicine, East Lansing, USA; 2 Biomedical Sciences, Ohio University Heritage College of Osteopathic Medicine, Dublin, USA; 3 Anthropology, Michigan State University College of Social Science, East Lansing, USA; 4 Physiology, Lyman Briggs College, Michigan State University, East Lansing, USA

**Keywords:** abdominal aortic aneurysms, anatomy dissection, cadaver case report, leriche syndrome, organ donor, pelvic congestion syndrome

## Abstract

This case study presents the gross anatomical findings encountered during the prosection of two anatomical donors, both with an abdominal aortic aneurysm (AAA) that were documented in their medical histories. Clots extended inferiorly through the aortic bifurcation and into the common iliac arteries. The severity and locations of the AAAs and the atherosclerosis of the iliac arteries in the donors are suggestive of Leriche syndrome. Additional findings on one donor, including ovarian cysts and the congestion of the left ovarian vein, are suggestive of pelvic congestion syndrome. The aim of this case study was to present the gross anatomical findings of AAAs and investigate the probable linkage between AAAs, Leriche syndrome, and pelvic congestion syndrome.

## Introduction

Abdominal aortic aneurysm (AAA)

Recent literature has suggested a probable correlation between AAAs and aortic dissection (AD) with pelvic congestion syndrome [1]. Defined as a “balloonlike dilation of an artery that frequently presents as a pulsatile mass”, aneurysms are increasingly common in the elderly and tend to enlarge over time [2]. The abdominal aorta is a common location of aneurysm formation due to the decrease in elasticity and degradation of elastic and collagen fibers [2,3]. Risk factors for developing an AAA include smoking, hypercholesterolemia, hypertension, and family history [4]. Male individuals are more likely to develop AAAs; however, female individuals are more likely to experience rupture [4].

Data suggest that small aneurysms will increase in size at a rate of 0.2-0.4 cm per year; however, larger increases may occur, especially as the AAA enlarges [5]. The risk of rupture increases as the mass gains size and with systemic hypertension [2,6,7], with a rapid expansion rate being the best predictor of rupture [3]. The risk of rupture is low in AAAs below 4 cm, while the risk of rupture is much higher for those larger than 5.5 cm [8]. Trials of patients with AAAs ranging in size from 4 cm to 5.4 cm in diameter have suggested that early surgical repair has no benefit in terms of survival compared to frequent monitoring [6].

Due to a wider diameter and a higher tension in the vessel wall, aneurysms typically first appear in the aorta [7], and almost all AAAs are located in the infrarenal portion between the renal arteries and aortic bifurcation [2]. A normal adult abdominal aorta measures approximately 1.8 cm in diameter, with a diameter greater than 3.5 cm typically classified as “aneurysmal” [2].

Aortoiliac occlusive disease (AOD)

AOD, also referred to as Leriche syndrome, is a type of peripheral artery disease caused by severe atherosclerosis and occlusion of the abdominal aorta and common iliac arteries. Although there is variation in literature, AOD is classified by several types, including occlusion of the infrarenal abdominal aorta and common iliac arteries (Type I), which may also extend into the external iliac arteries (Type II) and the femoral and popliteal arteries (Type III) [9,10]. Symptoms of AOD have been described as extreme fatigue of lower extremities that appears quickly, bilateral atrophy of both lower extremities, lack of pulse in the leg and groin, and impotence [7]. Most often, pain in the lower limbs is alleviated with rest due to the occlusion of vessels that supply muscles in the gluteal region and thigh [10]. As this syndrome is often slow to progress, collateral arterial pathways may develop to maintain blood flow to the lower limb, which may delay these symptoms. 

Pelvic congestion syndrome (PCS)

PCS is noted to cause a significant amount of pelvic pain and varicosities, leading to a diminished quality of life, and can be difficult to diagnose [11,1]. The etiology of PCS varies; however, it involves the congestion of blood in the ovarian veins, most often occurring in the left ovarian vein due to its drainage into the left renal vein before returning to the inferior vena cava [11]. Recent research has demonstrated that dilation of the left ovarian vein and PCS are possible complications of abdominal aortic aneurysms and aortic dissections due to compression of the left renal vein [1]. Of note, such research did not observe any patients with dilation of the right ovarian vein.

This report examines the linkage between AAAs, PCS, and AOD on two prosected human cadavers (“donors”).

## Case presentation

AAAs were noted during the dissection of two anatomical donors: an 84-year-old female donor (Donor 1) and an 83-year-old male donor (Donor 2). In Donor 1, the AAA extended into the common, external, internal iliac, and femoral arteries. Additional clots were also noted in abdominal and pelvic veins, although these may be attributed to the embalming process. The donor also displayed ovarian cysts and dilation of the left ovarian veins. Donor 2 also presented with an AAA with a stent and clots extending into the common iliac arteries. This donor had no signs of dilation of abdominal or pelvic veins. The size and extent of the abdominal aortic aneurysms of both donors are suggestive of AOD of varying degrees. 

Donor 1

**Figure 1:**
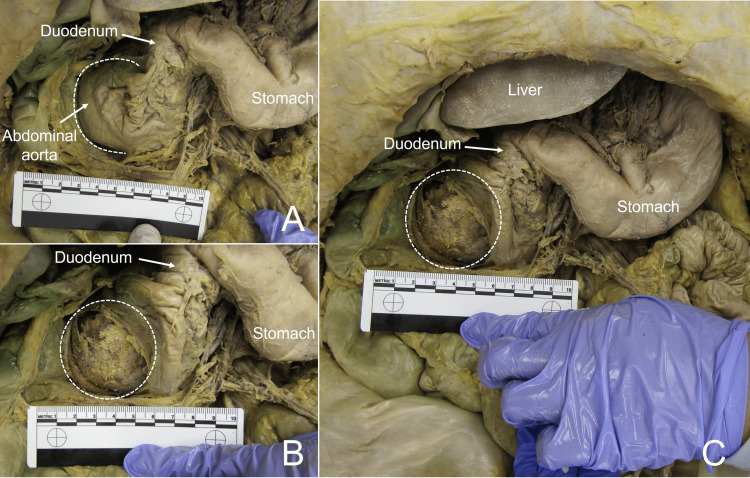
AAA in situ of Donor 1 before (Panel A) and after (Panels B, C) abdominal aorta was incised. Borders of the AAA visible during dissection are marked by dashed lines. AAA: abdominal aortic aneurysm

During the routine prosection of an 84-year-old female anatomical donor (Donor 1), a large AAA was noted. Due to the large size of the AAA, it originally appeared to be displacing the proximal duodenum laterally to the right (Figure 1). After further dissection, the borders of the AAA were identified. The AAA extended superiorly from the level of the left renal vein to the bifurcation of the abdominal aorta inferiorly, approximately 10 cm in length. The AAA clot further extended into the common iliac, external iliac, internal iliac, and femoral arteries. The AAA was found to be deviating the abdominal aorta to the right causing displacement and compression of the inferior vena cava. 

**Figure 2:**
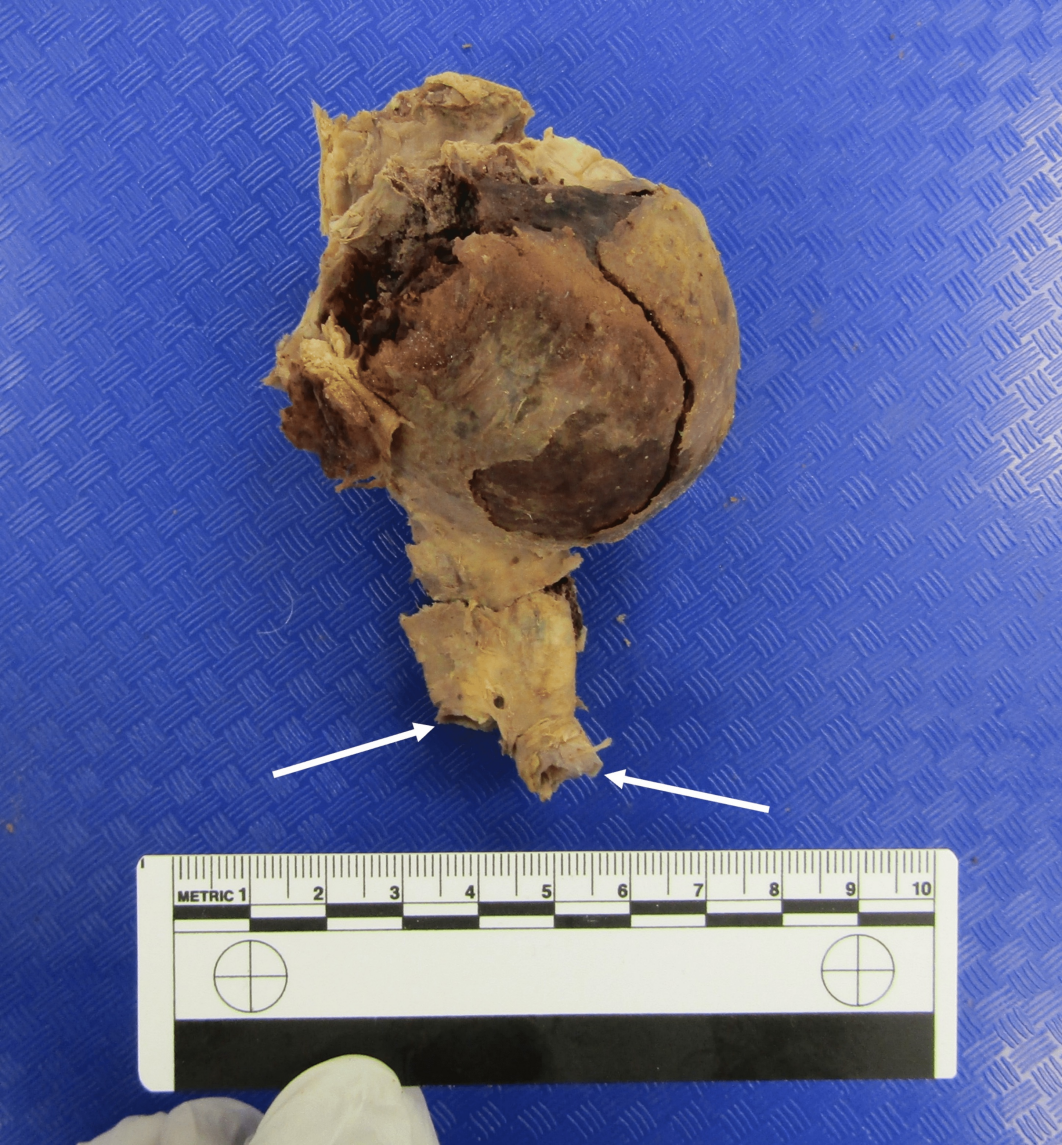
Thrombus removed from Donor 1. Arrows mark formations dissected from the common iliac arteries.

After removal, the thrombus was noted to be a large circular mass, measuring 10 cm (superior-inferior), 6 cm (medial-lateral), and 5.8 cm (anterior-posterior) (Figure 2).

**Figure 3:**
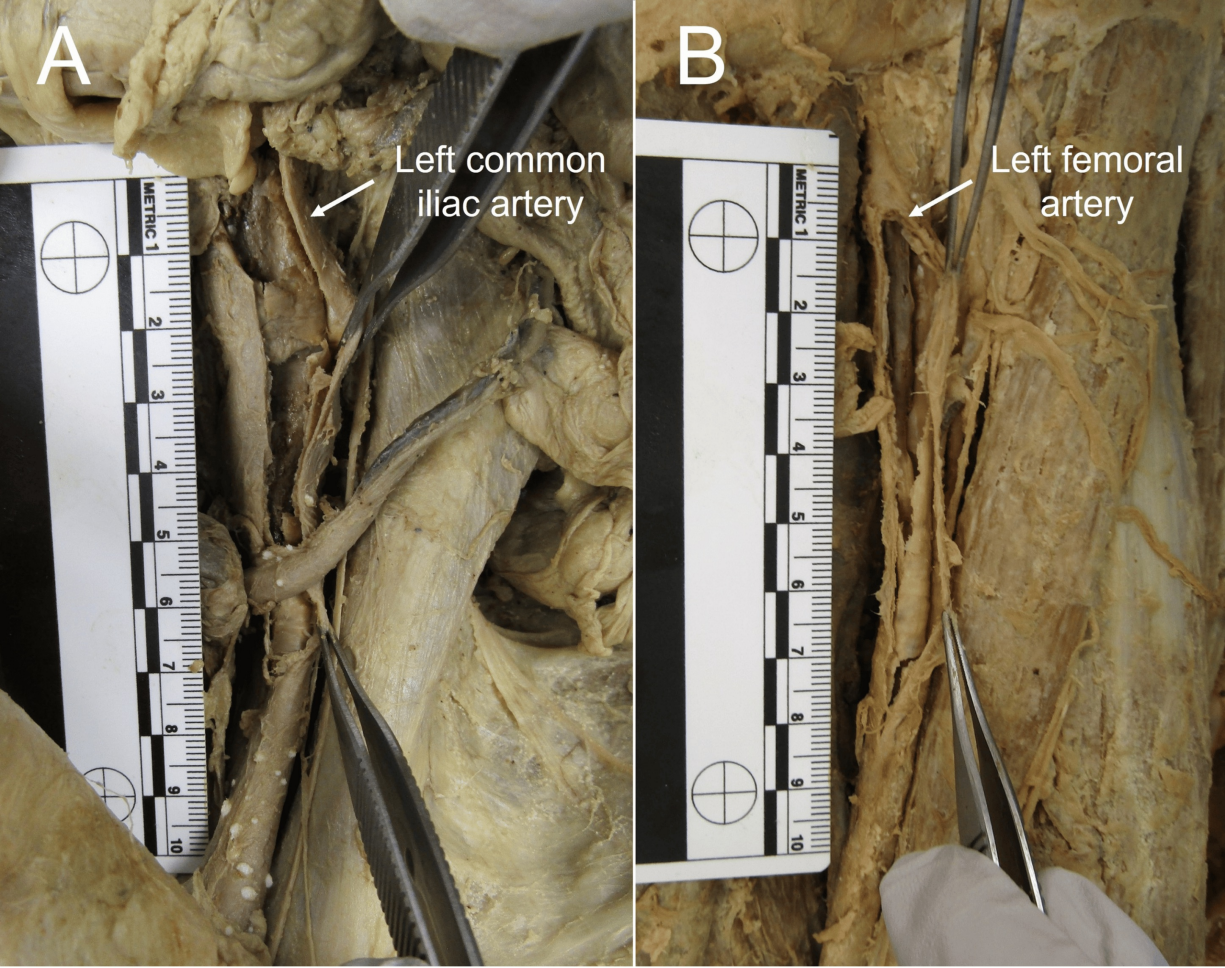
Panel A: Clot extended into left common and external iliac arteries. Panel B: Clot extended into left femoral artery.

Smaller portions of the AAA remained lodged within the common iliac arteries. Larger clots also extended into the external and internal iliac arteries and smaller clusters of clots extended into the femoral arteries (Figure 3). 

**Figure 4:**
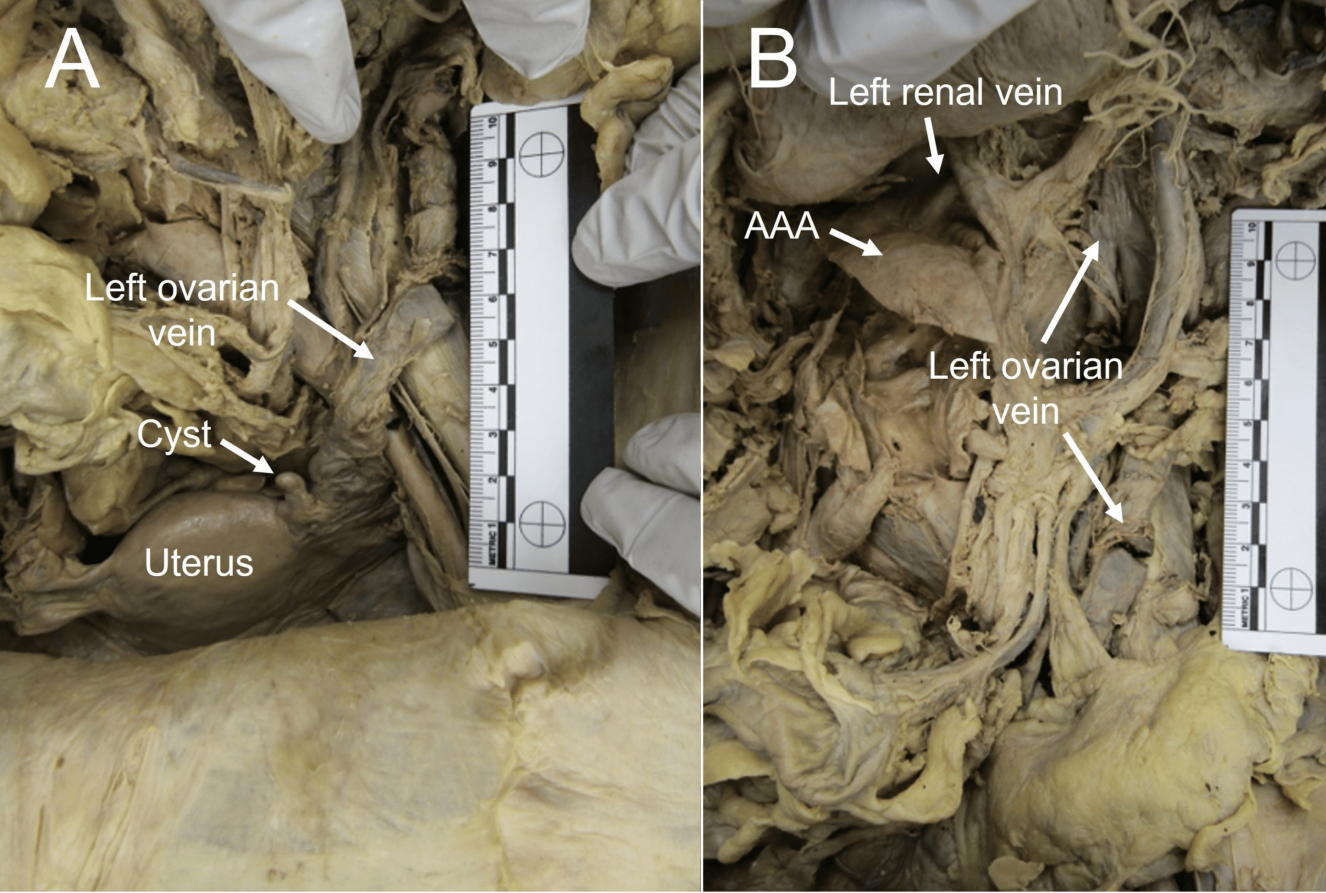
Left ovarian vein and ovarian cysts of Donor 1 (Panel A), with the left ovarian vein draining to the left renal vein (Panel B).

Upon further investigation of the abdomen and pelvis of the donor, more abnormalities were noted, including additional atherosclerosis and dilation of the left ovarian vein (Figure 4B) and multiple ovarian cysts (Figure 4A). The left renal and right ovarian veins were identified but did not appear to be dilated or contain clots. These additional gross anatomical findings are consistent with PCS and AOD.

Donor 2

**Figure 5:**
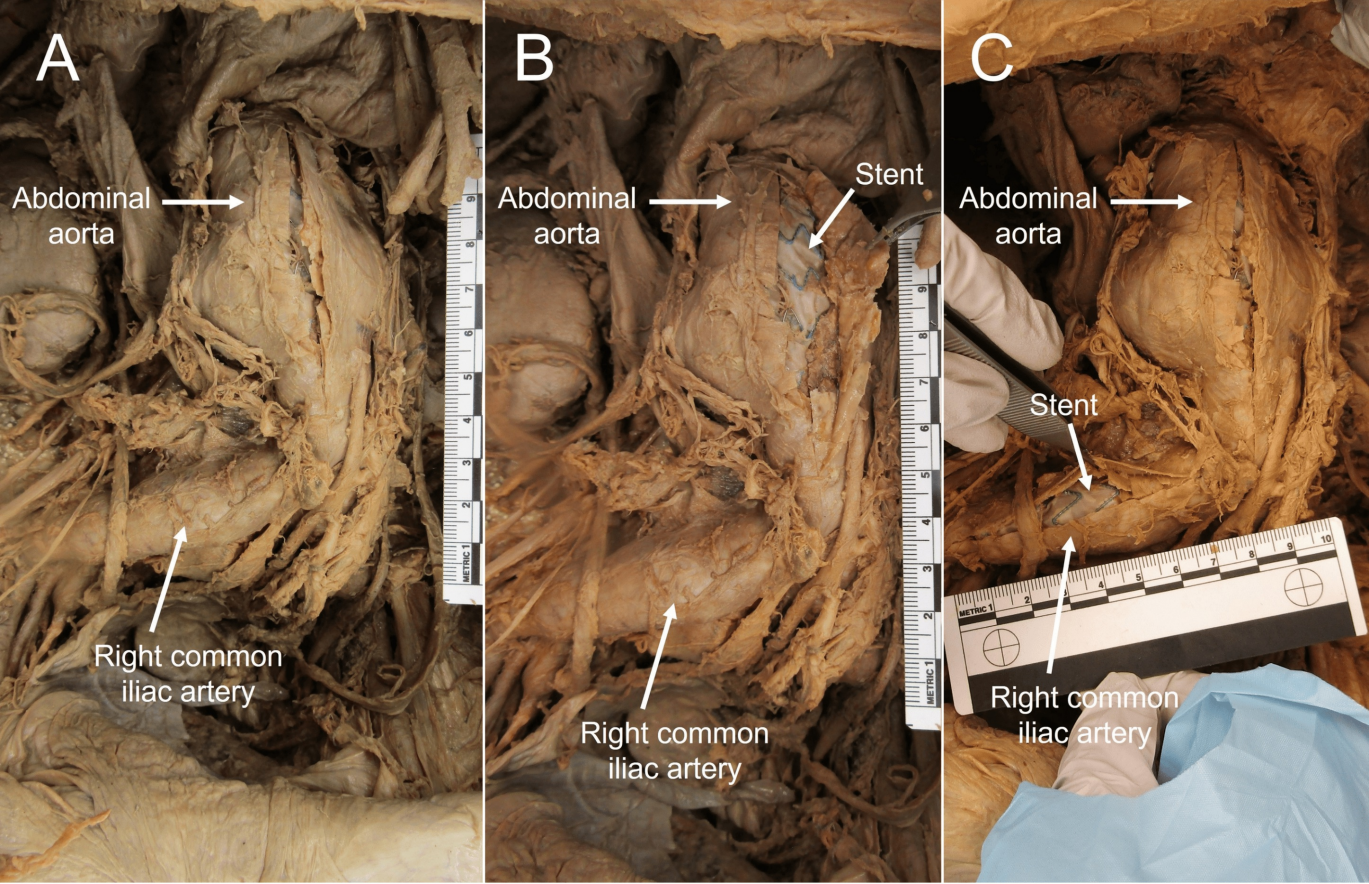
AAA and dilated right common iliac artery of Donor 2 (Panel A): the incised abdominal aorta (B) and incised right common iliac artery (Panel C) are reflected to show stent AAA: abdominal aortic aneurysm

During the routine prosection of an 83-year-old male anatomical donor (Donor 2), a large AAA was discovered (Figure 5A, 5B). The abdominal aorta was opened, and a stent was noted (Figure 5B). The AAA extended superiorly from the left renal vein to 2 cm superior to the bifurcation of the abdominal aorta inferiorly, approximately 9 cm in length (superior-inferior), 6 cm (medial-lateral), and 4 cm (anterior-posterior).

**Figure 6:**
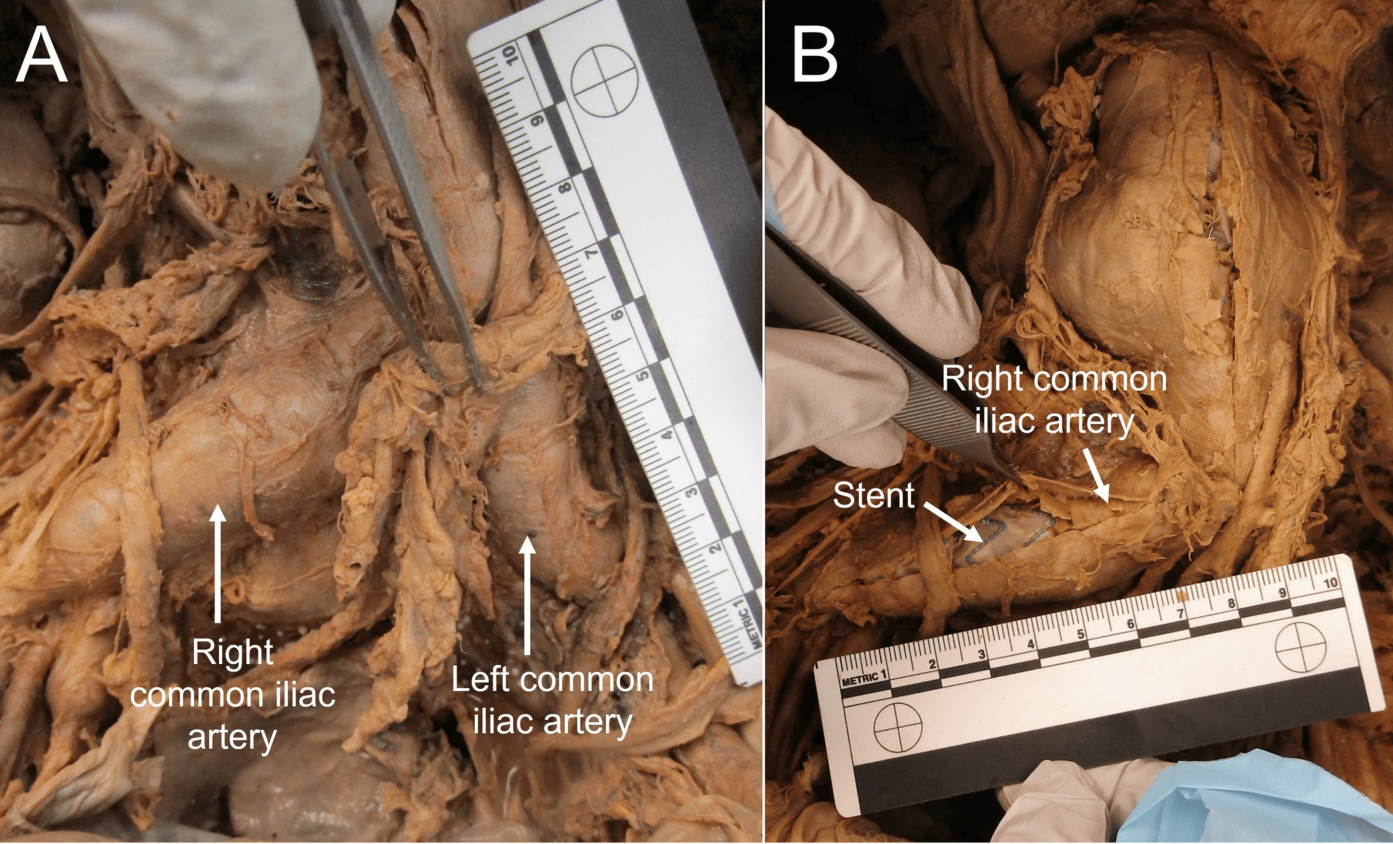
Panel A: Dilated common iliac arteries of Donor 2. Panel B: Incised and reflected right common iliac artery to show stent.

Additional clots extended through the distal aorta, the aortic bifurcation, and into the common iliac arteries (Figure 6A, 6B). This donor also exhibited a variation in which the left renal vein passed posterior to the abdominal aorta. However, no dilation of the renal veins was noted. These additional gross anatomical findings are consistent with the early onset of Leriche syndrome.

## Discussion

The limited medical history available for Donor 1 included a history of Alzheimer’s dementia, lung mass, and an AAA with no evidence of leak or rupture. The cause of death was listed as lung cancer. The limited medical history available for Donor 2 included a history of hypercholesterolemia, hypertension, bladder carcinoma, coronary arteriosclerosis, peptic ulcer, chronic obstructive pulmonary disease (COPD), dementia, and a chronic AAA. There was a surgical history of transurethral resection of the prostate, bilateral hip replacement, and multiple heart bypass surgeries. The cause of death was listed as shock, with a secondary cause of hypertension.

AAAs can either be asymptomatic or associated with a range of symptoms dependent on location [2,12,3]. In Donor 1, the aneurysm was noted to be compressing neighboring structures, especially the duodenum, and may have, therefore, been producing associated symptoms, such as abdominal pain. If AAAs produce symptoms, the most common is pain in the hypogastric region or lower back [3].

The AAAs in both donors extended almost the entire length of the infrarenal abdominal aorta. The AAA of Donor 1 extended from the left renal vein to the aortic bifurcation and inferiorly into the common, external, and internal iliac and femoral arteries. Dilation of the left ovarian veins was noted; however, no dilation was noticed in the right ovarian veins. It was unclear as to why dilation was present only on the left. The AAA of Donor 2 extended from the left renal vein to approximately 2 cm superior to the aortic bifurcation. Smaller clots extended into the distal abdominal aorta and into the common iliac arteries. The clots in the common iliac arteries dilated the arteries to about 2 cm in width. Both donors were only dissected on their anterior aspects; therefore, it is unknown if any possible thromboses were present in the distal femoral and popliteal arteries. Although not reported in the donor’s medical histories (listed above), the presentation of the clots is consistent with Type III AOD in Donor 1 and Type I AOD in Donor 2.

The pathologies noted on Donor 1 also support a linkage between AAAs and PCS. The occlusion of the inferior vena cava and congestion of primarily the left ovarian vein is evident, and multiple small ovarian cysts are visible. This is consistent with previous research that also noted only dilation of the left ovarian vein [1]. This same linkage was not noted in Donor 2. 

## Conclusions

Although the AAAs in both individuals were diagnosed during life, it is unknown what symptoms, if any, were observed. Additionally, symptoms associated with the above-mentioned conditions could have gone unreported due to the debilitating effects of dementia. While the nature of cadaveric dissection limits the ability to confirm possible diagnoses beyond what was provided in a medical history, the anatomical abnormalities observed in this case study are consistent with AOD for both donors and PCS for Donor 1. These findings, therefore, support the consideration of both conditions and their associated symptoms as potential complications in individuals with an AAA and promote an awareness of how such disruption from an AAA could progress over time.
